# Inferring Cell Subtypes and LncRNA Function by a Cell-Specific CeRNA Network in Breast Cancer

**DOI:** 10.3389/fonc.2021.656675

**Published:** 2021-04-27

**Authors:** Xin Chen, Jing Xu, Feng Zeng, Chao Yang, Weijun Sun, Tao Yu, Haokun Zhang, Yan Li

**Affiliations:** ^1^ School of Automation, Guangdong University of Technology, Guangzhou, China; ^2^ Department of Oncology, Changhai Hospital, The Naval Military Medical University, Shanghai, China; ^3^ Guangdong Key Laboratory of IoT Information Technology, School of Automation, Guangdong University of Technology, Guangzhou, China; ^4^ Department of Oncology, The First Affiliated Hospital of Nanjing Medical University, Nanjing, China; ^5^ Department of Bioinformatics, School of Biomedical Engineering and Informatics, Nanjing Medical University, Nanjing, China

**Keywords:** cell-specific network, ceRNA, estrogen regulation, lncRNA, subtype

## Abstract

Single-cell RNA sequencing is a powerful tool to explore the heterogeneity of breast cancer. The identification of the cell subtype that responds to estrogen has profound significance in breast cancer research and treatment. The transcriptional regulation of estrogen is an intricate network involving crosstalk between protein-coding and non-coding RNAs, which is still largely unknown, particularly at the single cell level. Therefore, we proposed a novel strategy to specify cell subtypes based on a cell-specific ceRNA network (CCN). The CCN was constructed by integrating a cell-specific RNA-RNA co-expression network (RCN) with an existing ceRNA network. The cell-specific RCN was built based on single cell expression profiles with predefined reference cells. Heterogeneous cell subtypes were inferred by enriching RNAs in CCN to the estrogen response hallmark. Edge biomarkers were identified in the early estrogen response subtype. Topological analysis revealed that NEAT1 was a hub lncRNA for the early response subtype, and its ceRNAs could predict patient survival. Another hub lncRNA, DLEU2, could potentially be involved in GPCR signaling, based on CCN. The CCN method that we proposed here facilitates the inference of cell subtypes from a network perspective and explores the function of hub lncRNAs, which are promising targets for RNA-based therapeutics.

## Introduction

The incidence of breast cancer has increased at a rate of 0.3% per year from 2012 to 2016 in the United States, largely because of the rising rates of local stage and hormone receptor-positive disease (1). As estrogen plays a predominant role in breast cancer, understanding the mechanisms of estrogen regulation holds profound significance in breast cancer research and treatment. The transcriptional regulation of estrogen receptor (ER) is an intricate network of signaling and functional processes that is still largely unknown at the single cell level. Recently, the intra-cell line heterogeneity of breast cancer has been comprehensively characterized through single-cell RNA sequencing (scRNA-seq), revealing transcriptomic subpopulations within cell lines (2). Therefore, investigating the heterogeneity of estrogen regulation at the single cell level could shed more light on estrogen mechanisms and potential breast cancer therapeutics.

As an active metabolite of estrogen, 17*β*-estradiol (E2) is essential for both normal breast cells and malignant breast cancer cells. Zhu et al. performed scRNA-seq on estrogen receptor alpha positive breast cancer cells stimulated by E2. Their research revealed a dynamic transcriptional network in which estrogen signaling promotes breast cancer cell survival and growth by mediating a metabolic switch (3). They also provided valuable data resources to explore the heterogeneous response of cells from the same cell line upon estrogen stimulation.

Dai et al. proposed a probability theory-based method to construct a cell-specific network for individual cells (4), which innovatively characterized each cell from the perspective of a ‘stable’ gene network rather than ‘unstable’ gene expression. This prompted us to propose a novel strategy to characterize single cells from the perspective of networks. The RNAs interact in a complicated manner within cells. For example, RNA functions as microRNA (miRNA) sponges by competitively binding to the same miRNA, reducing the repression or degradation effect of the miRNA on the target genes. These RNAs are competing endogenous RNAs (ceRNAs). Evidence from studies indicates that long non-coding RNAs (lncRNAs) act as ceRNAs to compete with miRNAs with mRNAs. For example, *NEAT1* was reported to serve as a ceRNA of *ZEB1*, which competes with miR-448 in breast cancer (5). *PTEN*, a well-known tumor suppressor, regulates *MALAT1* expression by potentially sponging oncogenic miRNAs, including *miR-17* and *miR-20a* in breast cancer (6). Therefore, it is adequate for the characterization of single cells from the viewpoint of the ceRNA network. Wang et al. applied the cell-specific network method developed by Dai et al. and integrated public ceRNA regulations to build a database named LnCeCell, which comprised the predicted lncRNA-associated ceRNA networks at single-cell resolution (7). In this study, we aimed to investigate cell heterogeneity upon estrogen stimulation, from the perspective of the ceRNA network.

Liu et al. developed a sample-specific network (SSN) method to construct a personalized network for individual patients, based on the expression profile of these patients (8). Inspired by the SSN method, we designed a novel strategy to construct a cell-specific ceRNA network (CCN) by integrating a cell-specific RNA–RNA co-expression network (RCN) with an existing ceRNA network. The cell-specific RCN was first constructed from single cell expression profiles with the aid of predefined reference cells, provided by the SSN method. After incorporating public ceRNA networks into the RCN, the CCN was obtained. To dissect the heterogeneity of the cell response to estrogen, RNAs in CCN were enriched with estrogen response hallmarks. The edge biomarkers for the early estrogen response subtype were also identified in the CCN; *NEAT1* had high average degree among the early response cells, and ceRNA survival analysis indicated that NEAT1 and its ceRNAs could predict patient survival. Moreover, we inferred the function of another hub lncRNA, *DLEU2*, which might be involved in GPCR signaling, based on both Gene Ontology (GO) and REACTOME pathways. In summary, we established a novel method to construct a CCN and provide single-cell network-related insights into estrogen regulation in breast cancer.

## Materials and Methods

### Data Pre-Processing

We downloaded the scRNA-seq data from Gene Expression Omnibus (GEO) (accession number: GSE107858). Following the filtering process described in the paper (3), we performed further analysis on 84 MCF-7 cells. RNAs with fragments per kilo base of transcript per million reads mapped (FPKM) >1 in at least 25% (84 × 0.25 = 21) of the cells were used for further analysis. The filtering parameter is referred to the paper (9). The dataset GSE107863 for T47D was an independent validation cohort to support the findings obtained using MCF-7 cells.

### CeRNA Network From starBase

The ceRNA network was downloaded (10) (http://starbase.sysu.edu.cn/) using the Web API. The ceRNAs for all mRNAs, lncRNAs, and pseudogenes were downloaded using default parameters. The ceRNA network contained 308,266 ceRNA pairs composed of 18,942 RNAs. The complete information is presented in **Table S1**.

### Gene Sets for Markers

We obtained the known cancer-related genes from the Cancer Gene Census (CGC) database (http://cancer.sanger.ac.uk/cosmic/census), which contains 576 genes (11). Other 876 cancer-related genes were also downloaded from the Genetic Association Database (GAD) database (http://geneticassociationdb.nih.gov/).

Functional gene sets “HALLMARK_ESTROGEN_RESPONSE_EARLY” and “HALLMARK_ESTROGEN_RESPONSE_LATE” were downloaded and extracted from the hallmark gene sets of MSigDB (https://www.gsea-msigdb.org/gsea/msigdb/, v7.2). REACTOME pathways and biological processes information of GO was also downloaded from MSigDB.

We downloaded the transcript annotation from Ensembl and obtained 215,307 annotations. The transcript ID, transcript type, and HUGO Gene Nomenclature Committee (HGCN) symbols were downloaded from Ensembl. Further, the annotations whose transcript type belonged to “lincRNA” or “antisense” were extracted as the lncRNAs. We obtained a total of 1,794 lncRNAs. In addition, we also downloaded the lncRNA annotation file lncipedia_5_0_hg19.gtf (full database) from LNCipedia (12). Considering that some lncRNAs had alternative names, we extracted the Ensembl ID, gene_alias and gene_id for each lncRNA. The lncRNA information from either Ensembl or LNCipedia was used to annotate the lncNRAs.

ER is the most important hormone receptor in breast cancer. We also screened the differentially expressed genes (DEGs) between ER-positive and ER-negative patients from public cohorts and denoted as ER_DEGs markers. The raw read counts for breast cancer was downloaded from The Cancer Genome Atlas (TCGA). The R package DESeq2 (13) was used for differential analysis. 22,946 DEGs were identified with adjusted p <0.05. The Z-score scaled expression profile of Molecular Taxonomy of Breast Cancer International Consortium (METABRIC) was also downloaded. T-test was used as the statistical method to calculate the p value of gene expression difference between ER+ *vs* ER− samples. The p value was then adjusted by fdr method using the R package fdrtool (14). As a result, we obtained 2,951 DEGs with fdr adjusted p <0.05.

### Constructing a CCN Based on Reference Cells

The SSN method was developed by Liu et al. to construct a personalized network for individual patients based on their expression profiles (8). Briefly, a reference network can be constructed using the Pearson correlation coefficient (PCC) between molecules based on the expression data of the reference samples. After a new sample is added to the reference samples, the perturbed network can be similarly constructed. Then, the differential network is constructed by the edges with significantly changed correlation between the reference and perturbed networks.

The changed correlation follows a new type of distribution, which is called the “volcano distribution”. The tail areas of this distribution are similar to those of the normal distribution based on the Kolmogorov–Smirnov test with random sampling. Therefore, the statistical hypothesis Z-test was used to evaluate the significance level of the changed correlation because of the central limit theorem (15).

**Figure 1:**
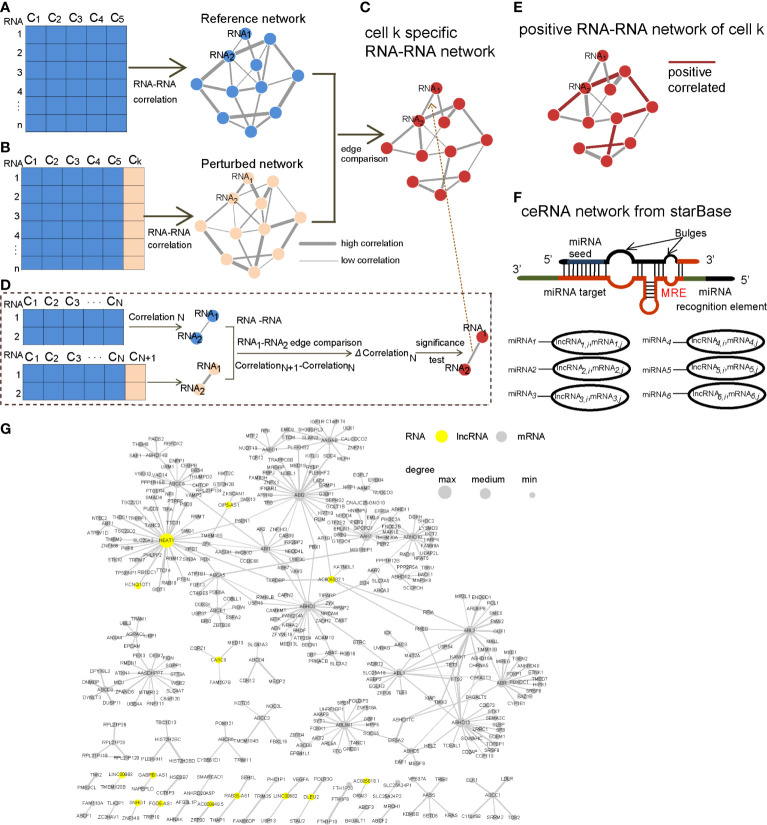
Workflow of constructing the CCN. **(A)** Reference cells were selected, and the corresponding RCN was constructed and referred to as “Reference network”. **(B)** One cell k was added to the reference cells, and the corresponding RCN was constructed and referred to as the “Perturbed network”. **(C)** Edges were compared between the “Reference network” and “Perturbed network” to obtain a cell k-specific RNA–RNA network. **(D)** One edge composing of two RNAs was compared and tested for its significance level, based on the differential correlation (Δ*Correlation_N_*). **(E)** Only the positive edges in the cell k-specific RNA–RNA network were candidate edges in the CCN. **(F)** We downloaded the ceRNA network from starBase. **(G)** An example of a CCN. The yellow nodes represent lncRNAs, and node size is proportional to its degree in the CCN.

Liu et al. selected 8–17 normal samples as reference samples to construct an SSN. They also ensured that the SSN is robust and stable for the different reference sample sizes. Inspired by the SSN method, 20 MCF-7 cells captured at 0 h were selected as the reference cells in this study (**Figure 1A**). The reference network was constructed using the PCC. The RNA–RNA correlation was deemed significant with a p-value <0.05. For cells at 3, 6, or 12 h, we added one cell to the reference cells and recalculated the RNA–RNA correlation (**Figure 1B**). We retained a correlation network, named the perturbed network, containing significant RNA–RNA relationships with a p-value <0.05.

Next, we compared the significant RNA–RNA interactions in the perturbed network and reference network to keep only the edges with significantly changed correlations (**Figure 1C**). **Figure 1D** shows how to test the significance of the changed correlation (Δ*Correlation*_*N*_) between a pair of RNAs. According to the SSN theory proposed by Liu et al. (8), the differential correlation followed a normal distribution, and the significance could be evaluated based on the Z-test.

$$\text{Z}=\frac{{\Delta \text{Correlation}}_{\text{N}}-\mu_{\Delta \text{Correlation}}}{\sigma_{\Delta \text{Correlation}}}=\frac{{\Delta \text{Correlation}}_{N}}{\frac{1-{\text{Correlation}}_{\text{N}}^{2}}{\text{N}-1}},$$

where *N* is the number of reference cells.

From the expression perspective, the ceRNAs were positively correlated. Therefore, we considered only the positive and significant differential RNA–RNA interactions as candidate ceRNAs (**Figure 1E**). The ceRNA network from StarBase was further used to filter the ceRNA network to ensure its biological importance (**Figure 1F**).

### Functional Enrichment of Genes in the CCN

The hypergeometric test was used to evaluate whether the genes in the CCN were significantly enriched in functional gene sets.

$$\text{P}=\sum_{\text{x}\geq n}\frac{{\text{C}}_{N}^{\text{x}}˙{\text{C}}_{\text{M}-\text{m}}^{\text{m}-\text{x}}}{{\text{C}}_{M}^{\text{m}}}$$

where *M* is the total number of genes in the background network, *N* is the number of genes in a functional gene set, *m* is the number of genes in the CCN, and *n* is the number of CCN genes shared by the functional gene set.

### Topology Analysis of CCN

The R package igraph was used to calculate the topological features of RNAs in the CCN (R 4.0.2). The degree of RNA is the number of direct neighbors in the ceRNA network. RNAs with a high degree can be termed as hub RNAs, which play a pivotal role in maintaining the ceRNA–ceRNA relationships within CCN. The betweenness of RNA *i* can be calculated with the formula

$$\frac{{\text{B}}_{i}=\sum_{\text{s}\neq \text{i}\neq i}\delta_{\text{st}}(\text{i})}{{\text{d}}_{\text{st}}},$$

where RNA *s* and *t* are RNAs in the CCN different from RNA *i*, *d_st_* represents the number of the shortest paths from *s* to *t*, and *δ_st_(i)* is the number of the shortest paths from *s* to *t* that *i* lies on. For RNA *s* and *t*, the ratio is the proportion of the shortest path that RNA *i* lies on. The sum of the ratios of all RNA pairs is the betweenness centrality of RNA *i*. The closeness coefficient is the average closeness of RNA *i* to other RNAs in the network. It is calculated as

$$\text{C}(\text{i})=\frac{1}{{\text{d}}_{i}}=\frac{\text{n}-1}{\sum_{\text{s}\neq \text{i}}{\text{d}}_{\text{si}}},$$

where *d_si_* represents the distance between RNA *i* and other RNAs.

### Survival Analysis of CeRNAs

A recently published paper (7) has provided a web tool, “ceRNA survival”, to perform survival analysis for ceRNA composed of lncRNA–miRNA–mRNA, based on The Cancer Genome Atlas (TCGA) datasets. The web tool was used to perform multivariate Cox regression analysis based on miRNA, mRNA, and lncRNA expression, without co-factors.

### Gene Set Enrichment Analysis

GSEA (16) was performed using Preranked utility implemented in the standalone version of the GSEA software (v 4.1.0). The RNA sequencing dataset of *DLEU2* knockdown was downloaded from the GEO database (https://www.ncbi.nlm.nih.gov/geo/), with accession number GSE162677. We ranked the genes according to the fold change in expression (FPKM.siDLEU2/FPKM.siNC). The fold change was log2 transformed before GSEA.

## Results

### CCN Construction Based on Reference Cells

The CCN was constructed following the workflow shown in **Figure 1** (more details in *Materials and Methods*). Briefly, the edges with significant differential correlation between the reference network (**Figure 1A**) and perturbed network (**Figure 1B**) were used to construct a cell-specific RCN (**Figure 1C**). miRNA targets are negatively regulated by miRNAs. RNAs competitively bind to the same miRNAs as ceRNAs. Chen et al. generated a ceRNA network for each subtype of breast cancer, based on the principle of positive co-expression and shared miRNAs (17). Therefore, the positive RCN appeared to be a candidate ceRNA, based on ceRNA theory (**Figure 1E**). The ceRNA network from starBase was further used to reduce false-positive ceRNA relations (**Figure 1F**). As an example, we have demonstrated the ceRNA network for the cell MCF-7 12 h (RHM266) in **Figure 1G**.

### Estrogen Receptor Alpha Co-Expressed With Known Marker Genes

**Figure 2:**
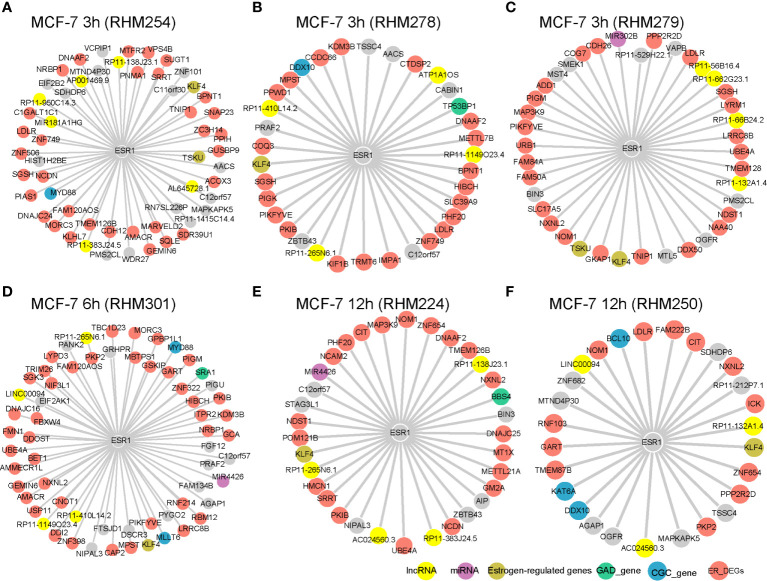
ESR1 interactors in cell-specific RCN. The yellow nodes represent lncRNAs, purple nodes represent miRNAs, green nodes represent GAD genes, blue nodes represent CGC genes, khaki nodes represent estrogen regulated genes, salmon nodes represent DEGs between ER+ *vs* ER− patients from either TCGA or METABRIC, and gray nodes represent genes with an unknown “biological” label. The RCN is shown for the **(A)** MCF-7 3 h (RHM254), **(B)** MCF-7 3 h (RHM278), **(C)** MCF-7 3 h (RHM279), **(D)** MCF-7 6 h (RHM301), **(E)** MCF-7 12 h (RHM224), and **(F)** MCF-7 12 h (RHM250) cells.

*ESR1* plays an important role in breast cancer. Therefore, we first examined its interactors in the cell-specific RCN, which is schematically shown in **Figure 1E**. We selected cells at 3, 6, and 12 h, in which *ESR1* had a degree larger than 25. The known estrogen-regulated genes (ERGs), such as *KLF4* (**Figures 2A–F**) and *TSKU* (**Figures 2A, C**), were significantly positively correlated with *ESR1*. Akaogi et al. reported high expression of *KLF4* in ER-*α*-positive patients. *KLF4* was found to bind to the DNA-binding region of ER-*α* and inhibit the binding of ER-*α* to estrogen response elements in the promoter regions (18). Known cancer-related genes from CGC, such as *MYD88* (**Figures 2A, D**), *DDX10* (**Figures 2B, F**), *MLLT6* (**Figure 2D**), *BCL10* (**Figure 2F**), and *KAT6A* (**Figure 2F**) also interacted with *ESR1*. The expression of MYD88 could be modulated in a single nucleotide polymorphism (SNP)- and estrogen-dependent fashion (19). Breast cancer-related genes from GAD were also identified as *ESR1* interactors, including *TP53BP1* (**Figure 2B**), *SRA1* (**Figure 2D**), and *BBS4* (**Figure 2E**). Low expression of TP53BP1 is associated with increased local recurrence in breast cancer patients treated with conserving surgery and radiotherapy (20). In addition to these protein-coding genes, *ESR1* was also co-expressed with non-coding RNAs such as *MIR302B* (**Figure 2C**) and *MIR4426* (**Figure 2E**). LncRNAs *MIR181A1HG* (**Figure 2A**), *ATP1A1OS* (**Figure 2B**), and *LINC00094* (**Figure 2D**) were also shown to cross-talk with *ESR1*. ER_DEGs frequently interacted with *ESR1* within individual cells (**Figures 2A–F**). These results indicate that the cell-specific RCN reflect the genes’ regulations of breast cancer.

We also constructed a cell-specific RCN based on the expression profiles of the T47D dataset. *ESR1* was also co-expressed with known marker genes, including known ERGs, cancer-related genes from CGC, ER-DEGs, and breast cancer-related genes from GAD (**Figure S1**).

The CCN was then constructed by integrating the RCN with the starBase ceRNA database, which was developed based on CLIP-Seq data. The average numbers of edges and lncRNAs in the CCN are shown in **Table 1**. The average number of edges decreased after E2 stimulation, from 882 edges (3 h) to 662 edges (12 h). Meanwhile, the average number of lncRNAs decreased from 16 (3 h) to 14 (12 h). In contrast, there were more than 1,600 edges on average in the CCN from the T47D dataset. However, on average, five lncRNAs were involved in the CCN.

**Table 1:** Average number of edges and lncRNAs in CCN.

Group	Average number of edges	Average number of lncRNAs
3 h	882	16
6 h	809	16
12 h	662	14

### Cell Subtypes Inferred by CCN

Cell type classification assumes high importance in single cell heterogeneity. Therefore, we defined cell subtypes by integrating CCN and the estrogen response hallmark. We retrieved estrogen early response and late response hallmarks from MSigDB. For each CCN, we extracted all the RNAs and performed a hypergeometric test to evaluate the extent of RNA enrichment occurring in these hallmark stages.

**Figure 3:**
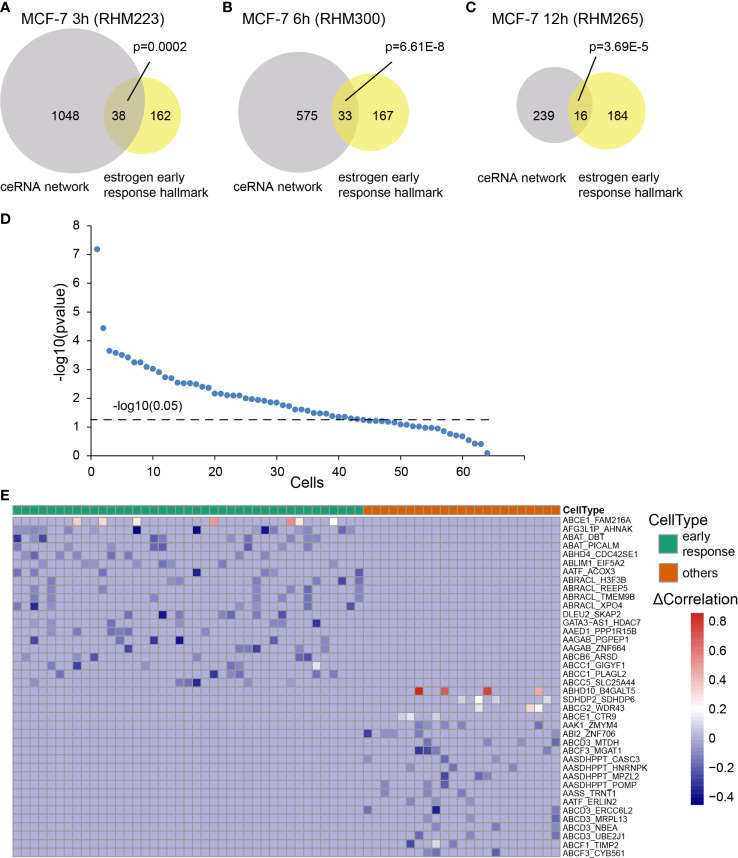
Cell subtypes inferred by the CCN. The RNAs in the CCN were enriched into estrogen early response hallmark, as determined by a hypergeometric test. We showed significant enrichment of RNAs in the CCN of the **(A)** MCF-7 3 h (RHM223), **(B)** MCF-7 6 h (RHM300), and **(C)** MCF-7 12 h (RHM265) cells to the early response hallmarks. **(D)** The minus log10 transformed p-value calculated by a hypergeometirc test for all cells at 3, 6, and 12 h. The dashed line represents the significance threshold, p = 0.05. **(E)** The heatmap of differential correlation (Δ*Correlation*) in all cells that were classified into two subtypes: early response cells *vs.* others. Blue represents loss of correlation in the “Perturbed network”, while red refers to the gain of correlation in the “Perturbed network”.

We selected one CCN at each time point as an example. The CCN was significantly enriched in the early response hallmark for MCF-7 3 h (RHM223, **Figure 3A**, p = 0.0002), MCF-7 6 h (RHM300, **Figure 3B**, p = 6.61E-8), and MCF-7 12 h (RHM265, **Figure 3C**, p = 3.69E-5). Among all 64 cells (3, 6, and 12 h), 41 were enriched in the estrogen early response hallmark (**Figure 3D**, p < 0.05). Three out of 64 cells were enriched in the estrogen late response hallmark (**Figure S2**). Similarly, the cells T47D 3 h (T47D_3 h_2B6), T47D 6 h (T47D_6 h_2H8), and T47D 12 h (T47D_12 h_D8) were enriched in the early response hallmark (**Figures S3A–C**). In the T47D dataset, seven cells were classified as early response cells (**Figure S3D**).

We further classified all 64 cells into two subtypes: early response ones and others. Traditionally, the nodes (RNAs in the network) were screened for biomarker identification. As a complex disease, breast cancer is induced by a set of dysregulated and synergetic genes rather than a single gene. Therefore, network biomarkers are more advantageous for characterizing disease states. Here, we explored the edge markers of both the cell subtypes. We selected the top 20 ceRNA–ceRNA relationships that only appeared in one cell subtype. From the heatmap, we could clearly distinguish the early response subtype from the other subtype (**Figure 3E**). A similar result is shown in **Figure S3E** for the T47 dataset.

### CeRNAs of Hub LncRNA Can Predict Patient Survival

Topological characterization of the CCN is crucial for identifying the pivotal genes that substantially contribute to gene regulation upon E2 stimulus. For all the CCNs, we analyzed the topological features, including degree, betweenness, and closeness coefficients. We focused on lncRNAs in the CCN with a high degree in the early response subtype. The top five lncRNAs are listed in **Table 2**. The average degree for all lncRNAs in the early response subtype is in **Table S3**.

**Table 2:** LncRNAs with top degree in early response subtype.

Official_Symbol	Average_degree
OIP5-AS1	5.36
NEAT1	5.14
DLEU2	4.06
GABPB1-AS1	3.43
DLEU1	2.77

**Figure 4:**
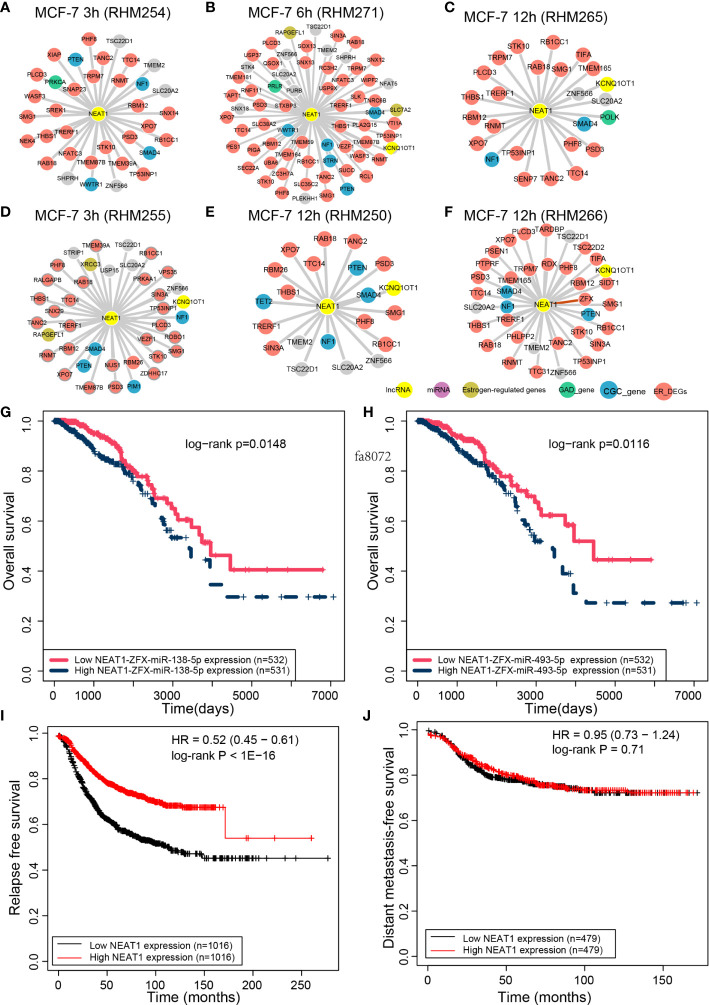
NEAT1 interactors in the CCN. The interactions between NEAT1 and its ceRNAs in the **(A)** MCF-7 3 h (RHM254), **(B)** MCF-7 6 h (RHM271), **(C)** MCF-7 12 h (RHM265), **(D)** MCF-7 3 h (RHM255), **(E)** MCF-7 12 h (RHM250), and **(F)** MCF-7 12 h (RHM266) cells. The survival analysis of NEAT1 and its ceRNA ZFX binding to **(G)** miR-138-5p and **(H)** miR-493-5p. The relapse-free **(I)** and metastasis-free survival analysis **(J)** performed by Kaplan–Meier Plotter for NEAT1.

The role of *NEAT1* in breast cancer has been widely investigated. It is also a hub lncRNA in the CCN of early response cells with an average degree as high as 5.14. Cells with a degree ≥20 for *NEAT1*were selected. *SMAD4* and *NF1*, the known cancer-related genes in the CGC database, are the common ceRNAs of *NEAT1* in all six CCNs (**Figures 4A–F**). Another CGC gene, *PTEN*, appears in five of these six ceRNA networks. *WWTR1*, a CGC gene, is the ceRNA of *NEAT1* in cell MCF-7 3 h (RHM254, **Figure 4A**) and MCF-7 6 h (RHM271, **Figure 4B**). The GAD genes *PRKCA*, *PRLR*, and *POLK* function as ceRNAs of *NEAT1* in cell MCF-7 3 h (RHM254, **Figure 4A**), MCF-7 6 h (RHM271, **Figure 4B**), and MCF-7 12 h (RHM265, **Figure 4C**), respectively. For cell MCF-7 3 h (RHM255, **Figure 4D**), we identified the ERGs *XRCC1* and *RAPGEFL1* as ceRNAs of *NEAT1*. For cell MCF-7 6 h (RHM271), ERGs *RAPGEFL1* and *SLC7A2* are ceRNAs of *NEAT1*. *TET2*, a CGC gene, is the ceRNA of *NEAT1* in cell MCF-7 6 h (RHM250, **Figure 4E**).

Among *NEAT1* ceRNAs in the six CCNs, we noted that the ceRNA–ceRNA relationship of *NEAT1* and *ZFX* in MCF-7 12 h (RHM266, **Figure 4F**) has been recently validated (21). According to a previous study (21), *NEAT1* and *ZFX* competitively bind to *miR-138-5p*. Next, we performed multivariate Cox regression analysis for the *NEAT1*–*ZFX*–*miR-138-5p* regulation axis using the “ceRNA survival” tool of LnCeCell (7). We divided all breast cancer patients from TCGA into two groups, based on the median expression value of *NEAT1*–*ZFX*–*miR-138-5p*. Patients with high *NEAT1*–*ZFX*–*miR-138-5p* expression had worse overall survival than those with low expression (**Figure 4G**). Moreover, we curated from starBase that *miR-493-5p* and *miR-513a-5p* are significantly shared by *NEAT1* and *ZFX*. Because the expression of *miR-513a-5p* is not available in the TCGA dataset of breast cancer, we tested the prognostic potential of *NEAT1*–*ZFX*–*miR-493-5p*. As shown in **Figure 4H**, the *NEAT1*–*ZFX*–*miR-493-5p* axis was also an unfavorable prognostic marker of breast cancer.

As *NEAT1* is one of the hubs in CCN, we further used the web tool Kaplan–Meier Plotter (https://kmplot.com/analysis) to perform relapse-free and metastasis-free survival analysis for *NEAT1*. Three probes from the microarrays were mapped to *NEAT1*. The mean expression of the probes was used for the survival analysis of *NEAT1*. High and low *NEAT1* expression levels were divided according to the median expression level. As shown in **Figure 4I**, *NEAT1* was a prognostic marker for breast cancer, based on relapse-free survival analysis. However, *NEAT1* was not a predictor of metastasis-free survival in breast cancer (**Figure 4J**).

We also constructed CCNs based on the T47D dataset. *NEAT1* is not a hub lncRNA in the CCNs of T47D cells. The top five lncRNAs in the early response cells are shown in **Table S2** for the T47D dataset. Topping the list is *PITPNA-AS1*. But there is no available siLncRNA dataset for *PITPNA-AS1*. Therefore, we focus on MALAT1, which has the second largest degree. *MALAT1* has been widely investigated for its role in breast cancer. Cells with a degree ≥10 for *MALAT1* were selected (**Figures S4A–C**). *MALAT1* interacts with known ERGs, cancer-related genes from CGC, ER-DEGs, and breast cancer-related genes from GAD, which is consistent with the results from the MCF-7 dataset. The ceRNA–ceRNA relationships of *MALAT1*–*ZFP36L2*, *MALAT1*–*PGRMC2*, and *MALAT1*–*PDS5B* were shared among the three cells (**Figures S4A–C**). The ceRNA survival analysis revealed that they were unfavorable prognostic markers for breast cancer (**Figures S4D–F**). Survival analysis of the hub lncRNA *MALAT1* demonstrated that *MALAT1* was a prognostic marker of relapse-free survival (**Figure S4G**) but not metastasis-free survival (**Figure S4H**).

### Function of the Hub LncRNA Can Be Inferred With CCN

Function prediction and interpretation of lncRNAs are important factors to dissect their biological mechanisms. Therefore, we tried to infer the function of the hub lncRNA *DLEU2*, which has not been characterized well in breast cancer.

The silencing or overexpression is a commonly used measure of lncRNA function inference. We searched the GEO database for RNA-sequencing datasets generated by siLncRNA or overexpression of *DLEU2*. As a result, we found that the dataset for siDLEU2 (GSE162677) matched our criteria.

Differential expression analysis is a commonly used method to explore the function of lncRNAs, especially for *in silico* experiments. The significantly affected biological functions associated with *DLEU2* expression could be theoretically identified based on functional enrichment of the genes affected by *DLEU2*. However, the dataset GSE162677 is generated from a cervical cell line. Thus, it is not suitable for the functional interpretation of *DLEU2* in estrogen regulation in breast cancer.

**Figure 5:**
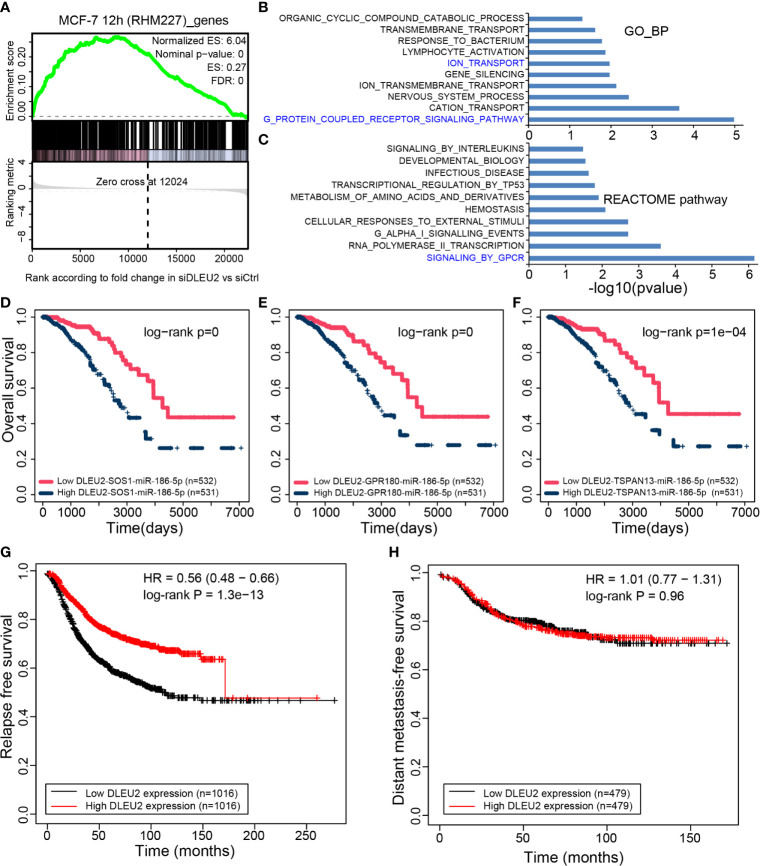
Function inference of DLEU2 *via* the CCN. **(A)** GSEA of RNAs in MCF-7 12 h (RHM227) cell to RNAs affected by siDLEU2. The top 10 functional terms from the **(B)** biological process of GO (GO_BP) and **(C)** REACTOME pathways enriched by RNAs in the CCN of MCF-7 12 h (RHM227) cell, as determined by the hypergeometric test. The interested terms are colored in blue. DLEU2 and its ceRNAs **(D)** SOS1, **(E)** GPR180, and **(F)** TSPAN13 have prognostic potential for breast cancer. The relapse-free **(G)** and metastasis-free survival analysis **(H)** performed by Kaplan–Meier Plotter for DLEU2.

In MCF-7 cells at 12 h (RHM227), *DLEU2* had the highest number of ceRNAs. GSEA was used to enrich RNAs in the CCN. The RNAs in RHM227 were significantly enriched in the up-regulated genes after siDLEU2 (**Figure 5A**), which indicates that the genes in the CCN may have similar expression changes after siDLEU2 in breast cancer. Therefore, we further predicted *DLEU2* function based on the genes in the CCN and used the hypergeometric test to explore the function of *DLEU2* by functional enrichment of RNAs in the CCN of RHM227. The functional terms of the GO and REACTOME pathways were downloaded from MSigDB (v7.2). The top 10 most significant biological processes from GO and pathways from REACTOME are shown in **Figures 5B, C**, respectively. The most significant GO and REACTOME pathway was GPCR signaling. It should be noted that the biological process “ION_TRANSPORT” was also significantly enriched by RNAs in the CCN of RHM227.

Next, we focused on the ceRNAs of *DLEU2* in the CCN of RHM227. *SOS1* is involved in GPCR signaling from both GO and REACTOME, while *GPR180* participates in GPCR signaling from GO. *TSPAN13*, a ceRNA of *DLEU2*, is also a known marker of late estrogen response. It synchronizes with other genes to facilitate ion transport. We retrieved the miRNAs shared by *DLEU2*, *SOS1*, *GPR180*, and *TSPAN13*. Multivariate Cox regression analysis of ceRNAs demonstrated their prognostic potential in breast cancer (**Figures 5D–F**). Furthermore, relapse-free and metastasis-free survival analysis using Kaplan–Meier Plotter (https://kmplot.com/analysis) revealed that *DLEU2* can predict relapse-free survival (**Figure 5G**) but not metastasis-free survival (**Figure 5H**).

To validate the possibility of inferring lncRNA functions *via* the CCN, we also predicted the function of *MALAT1*, based on the CCN of T47D cells. The dataset GSE110239 is an mRNA profile generalized by RNA-sequencing for the mammary tumor mouse model PyMT. Mouse genes were mapped to human gene symbols using the R package biomaRt. The genes in the cell T47D_6 h_2C12 was significantly enriched in the up-regulated genes after *MALAT1* KO (**Figure S5A**). Further functional analysis of RNAs in the CCN showed that they were enriched in the pathway of “fatty acid metabolism” (p = 0.015). The “fatty acid metabolism” has also been reported to be enriched by DEGs between BPA exposure and control in mouse liver. Several DEGs were key drivers, such as *Apoa2*, *Akr1c12*, and *Malat1* (22). This provides additional support for the function inference of lncRNAs *via* the CCN.

## Discussion

The scRNA-seq technique has become a powerful tool for the elucidation of intra-tumor and intra-cell line heterogeneity in breast cancer (2, 23). Estrogen regulation generally involves not only individual molecules but also molecular networks. Therefore, identifying the CCN upon E2 stimulus is crucial to elucidate the cellular heterogeneity of estrogen regulation at the system level. The CCN is directly constructed based on the single-cell gene expression profile to avoid the bias caused by subjective cluster information. Moreover, Dai et al. demonstrated that gene associations, rather than gene expression, can stably portray biological processes in individual cells (4). We merged ceRNA relations with a cell-specific RCN, which reduced the false-positive RNA–RNA associations.

*ESR1* is a pivotal regulator of breast cancer. From the cell-specific RCN, we found that *ESR1* interacted with known ERGs, such as *KLF4* and *TSKU*. Other known cancer-related genes from CGC and GAD were also significantly correlated with *ESR1*. In addition to these protein-coding genes, miRNAs (for example, *MIR302B* and *MIR4426*) and lncRNAs (for example, *MIR181A1HG*, *ATP1A1OS*, and *LINC00094*) were predicted to interact with *ESR1*. *MiR-302* (including *miR-302b*) sensitizes MCF-7 cells to adriamycin and mitoxantrone (24, 25). *LINC00094* has been reported as a super-enhancer-associated ce-lncRNA that promotes cell growth in esophageal squamous cell carcinoma (26). It should be noted that GSE107858 only performed polyA RNA sequencing, without miRNA or lncRNA sequencing. The miRNAs or lncRNAs in the expression profile come from the process of mapping reads to the reference genome.

Heterogeneous cell subtypes upon E2 stimulus were inferred by enriching RNAs in CCN to the estrogen response hallmark. The results showed that 68.7% (44/64) of the cells were responsive to E2 stimulation, and 93.2% (41/44) of them were early response cells. We then classified the cells into two subtypes: early response cells and the remaining cells. The correlation differences of the top 20 edges are shown for each subtype in **Figure 3E**. Among these edge markers, some gene components are not differentially expressed along the time series, which means that they cannot be identified by traditional differential analysis based on gene expression. Regarding the edges of *GATA3-AS1* and *HDAC7*, neither is a differentially expressed gene. However, the edge of *GATA3−AS1* and *HDAC7* had a significant correlation difference in several early response cells. *GATA3*–*AS1* has been reported to be involved in triple-negative breast cancer progression and immune escape by stabilizing the PD-L1 protein and degrading the *GATA3* protein (27). In contrast, the edge of *AATF* and *ERLIN2* showed no correlation difference in early response cells but had a correlation difference in other cells. Although *AATF* and *ERLIN2* are not DEGs, *AATF* silencing may be utilized to evoke apoptosis and regulate the expression of ERs in MCF-7 cells (28). *ERLIN2* has been reported to promote cell survival by regulating endoplasmic reticulum stress in breast cancer. Moreover, its regulation by *miR-410* is ER-dependent (29).

LncRNA-associated ceRNAs have been investigated in breast cancer. To explore such key lncRNAs and their ceRNAs, topological features such as degree were utilized to identify lncRNAs that function as hub nodes in the CCN. *NEAT1* is the top hub gene observed in the early response subtype, indicating its pivotal role in estrogen regulation. The ceRNAs contain ERGs or cancer-related genes. Intriguingly, *NEAT1* and its ceRNAs can also serve as prognostic markers for breast cancer, which further reveals that the constructed CCN has potential clinical applications.

CCN was used to predict lncRNA function. The RNAs in the CCN of one T47D cell were significantly enriched in the up-regulated genes after *MALAT1* KO (**Figure S5A**). Functional enrichment results implied that RNAs in the CCN participated in the pathway of “fatty acid metabolism”, which has also been reported to be associated with BPA exposure, mainly driven by RNAs including *MALAT1* (22). This provides evidence for the functional inference of lncRNA *via* the CCN. We noticed that the hub lncRNA *DLEU2* had not been functionally characterized well in breast cancer. Therefore, the public siRNA datasets of *DLEU2* were selected to infer the function of *DLEU2*. The GSEA results (**Figure 5A**) indicate the feasibility of the functional interpretation of lncRNAs *via* RNAs in the CCN. Functional terms from GO and REACTOME both demonstrated that *DLEU2* is involved in GPCR signaling. In addition, the ceRNAs of *DLEU2* can also predict patient survival in breast cancer. These results can facilitate the speculation of the biological functions of hub lncRNAs, which have not been characterized.

The current CCN method had several limitations. We used the gene expression profile in FPKM, which was biased when comparing gene expression among samples. We also did not consider the impact of inter-sample normalization on our results. In this study, we considered only the positive and significantly differential RNA–RNA interactions as candidate ceRNAs, in view of direct miRNA targets. Negative correlations are also important because they might be translated as indirect targets of miRNAs sponged by particular ceRNAs. Therefore, the anti-correlated and significantly differential RNA–RNA interactions were added to the candidate ceRNAs. As a result, the anti-correlation will increase the size of cell-specific RCN (**Figure S6** and **Table S4**) and CCN (**Figure S7** and **Table S4**), but do not consequentially increase the significance of CCN enrichment in estrogen early response hallmarks (**Figure S8**). Moreover, it does not affect the function inference of lncRNAs *via* CCN (**Figure S7**).

To conclude, we proposed a novel strategy for constructing a CCN by integrating reference cell-based cell-specific RCNs and public ceRNA networks. This CCN provides new insights into the inference of cell subtypes by incorporating functional gene set information. Hub lncRNAs in the early response subtype and their ceRNAs could be potential prognostic markers for overall survival and relapse-free survival. This CCN also provides a new perspective to infer the functions of uncharacterized hub lncRNAs, which are potential targets for RNA-based therapeutics.

## Data Availability Statement

The original contributions presented in the study are included in the article/**Supplementary Material**. Further inquiries can be directed to the corresponding author.

## Author Contributions

XC and JX conceived the study. YL supervised the project. FZ, CY, and HZ performed the computational analysis. XC and JX drafted the manuscript. WS, TY, YL, and HZ revised the manuscript. All authors contributed to the article and approved the submitted version.

## Funding

This work was financially supported in part by grants from the National Natural Science Foundation of China [Grant No. 62003094 and 82003615] and Natural Science Foundation of Guangdong Province [Grant No. 2018A0303130080 and 2019A1515011377].

## Conflict of Interest

The authors declare that the research was conducted in the absence of any commercial or financial relationships that could be construed as a potential conflict of interest.

## Abbreviation

ESR1, estrogen receptor alpha; RCN, RNA–RNA co-expression network; ceRNAs, competing endogenous RNAs; CCN, cell-specific ceRNA network; scRNA-seq, single-cell RNA sequencing; lncRNAs, long non-coding RNAs; E2, 17*β*-estradiol; ERGs, estrogen regulated genes; GO, Gene Ontology; ER, estrogen receptor; SSN, sample-specific networks; CGC, Cancer Gene Census; GAD, Genetic Association Database; METABRIC, Molecular Taxonomy of Breast Cancer International Consortium; TCGA, The Cancer Genome Atlas; DEGs, differentially expressed genes; GEO, Gene Expression Omnibus; FPKM, fragments per kilo base of transcript per million reads mapped.
