# Supplementary material for: Inferring Cell Subtypes and LncRNA Function by a Cell-Specific CeRNA Network in Breast Cancer
Source: Front Oncol. 2021 Apr 27;11:656675. doi: 10.3389/fonc.2021.656675 (PMC8111082; doi:10.3389/fonc.2021.656675)
Supplement: Supplementary file 1 [file Image_1.pdf]

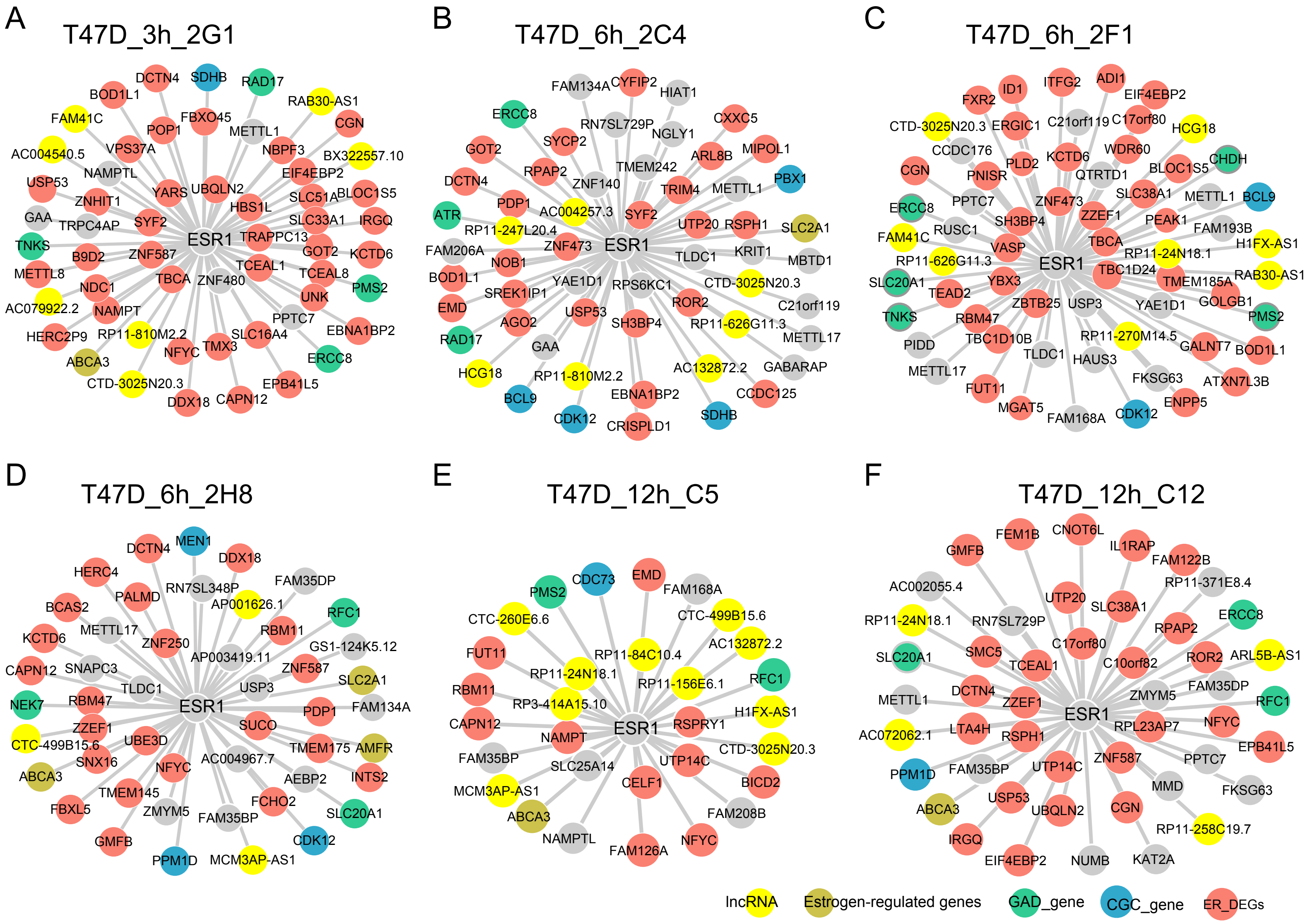

Figure S1. ESR1 interactors in cell-specific RCN of T47D cells. The yellow nodes represent lncRNAs, purple nodes represent miRNAs, green nodes represent GAD genes, blue nodes represent CGC genes, khaki nodes represent estrogen regulated genes, salmon nodes represent DEGs between ER+ vs ER- patients from either TCGA or METABRIC, and gray nodes represent genes with an unknown “biological” label. The RCN is shown for the (A) T47D_3h_2G1, (B) T47D_6h_2C4, (C) T47D_6h_2F1, (D) T47D_6h_2H8, (E) T47D_12h_C5, and (F) T47D_12h_C12.
