# Supplementary material for: Inferring Cell Subtypes and LncRNA Function by a Cell-Specific CeRNA Network in Breast Cancer
Source: Front Oncol. 2021 Apr 27;11:656675. doi: 10.3389/fonc.2021.656675 (PMC8111082; doi:10.3389/fonc.2021.656675)
Supplement: Supplementary file 2 [file Image_2.pdf]

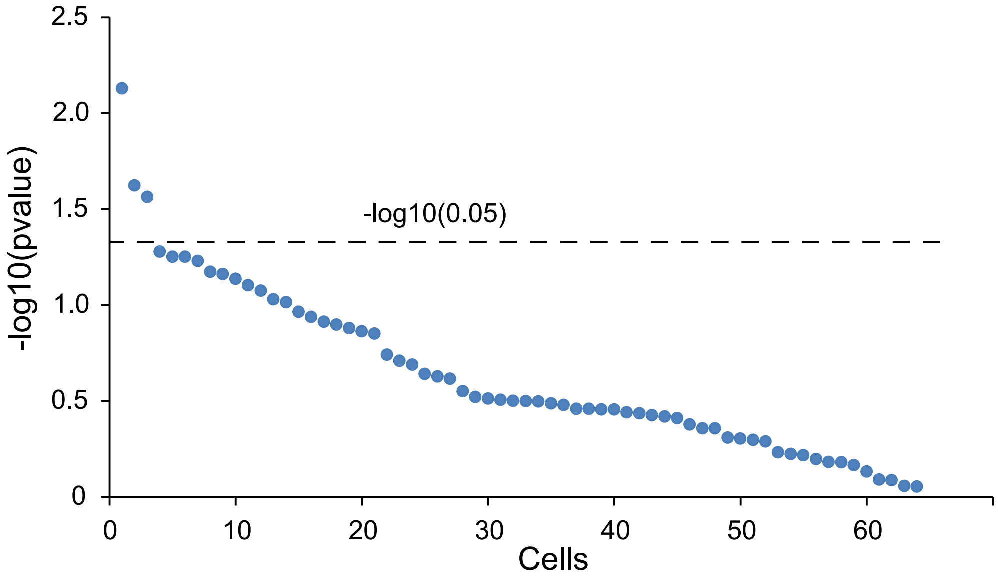

Figure S2. The RNAs in CCN were enriched into estrogen late response hallmark. The minus log10 transformed pvalue calculated by hypergeometirc test for all cells at 3h, 6h and 12h. The dashed line represents significance threshold p=0.05.
