# Supplementary material for: Inferring Cell Subtypes and LncRNA Function by a Cell-Specific CeRNA Network in Breast Cancer
Source: Front Oncol. 2021 Apr 27;11:656675. doi: 10.3389/fonc.2021.656675 (PMC8111082; doi:10.3389/fonc.2021.656675)
Supplement: Supplementary file 4 [file Image_4.pdf]

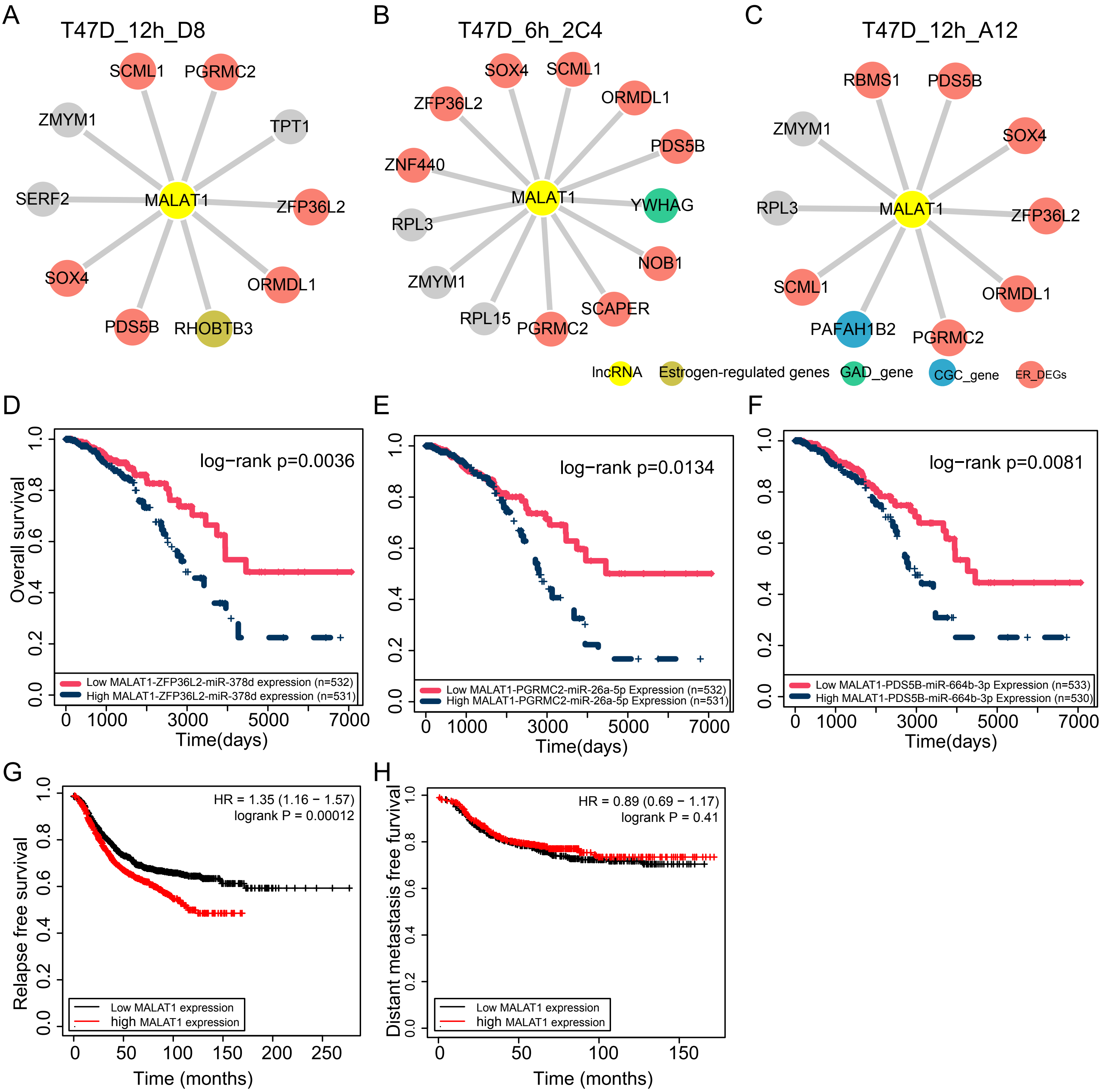

Figure S4. MALAT1 interactors in the CCN of T47D. The interactions between MALAT1 and its ceRNAs in the (A) T47D_12h_D8, (B) T47D_6h_2C4, (C) T47D_12h_A12 cells. The survival analysis of MALAT1 and its ceRNA ZFP36L2 binding to miR-378d (D), ceRNA PGRMC2 binding to miR-26a-5p (E), and ceRNA PDS5B binding to miR-664b-3p (F). The relapse-free (I) and metastasis-free survival analysis (J) performed by Kaplan-Meier Plotter for MALAT1.
