# Supplementary material for: Inferring Cell Subtypes and LncRNA Function by a Cell-Specific CeRNA Network in Breast Cancer
Source: Front Oncol. 2021 Apr 27;11:656675. doi: 10.3389/fonc.2021.656675 (PMC8111082; doi:10.3389/fonc.2021.656675)
Supplement: Supplementary file 5 [file Image_5.pdf]

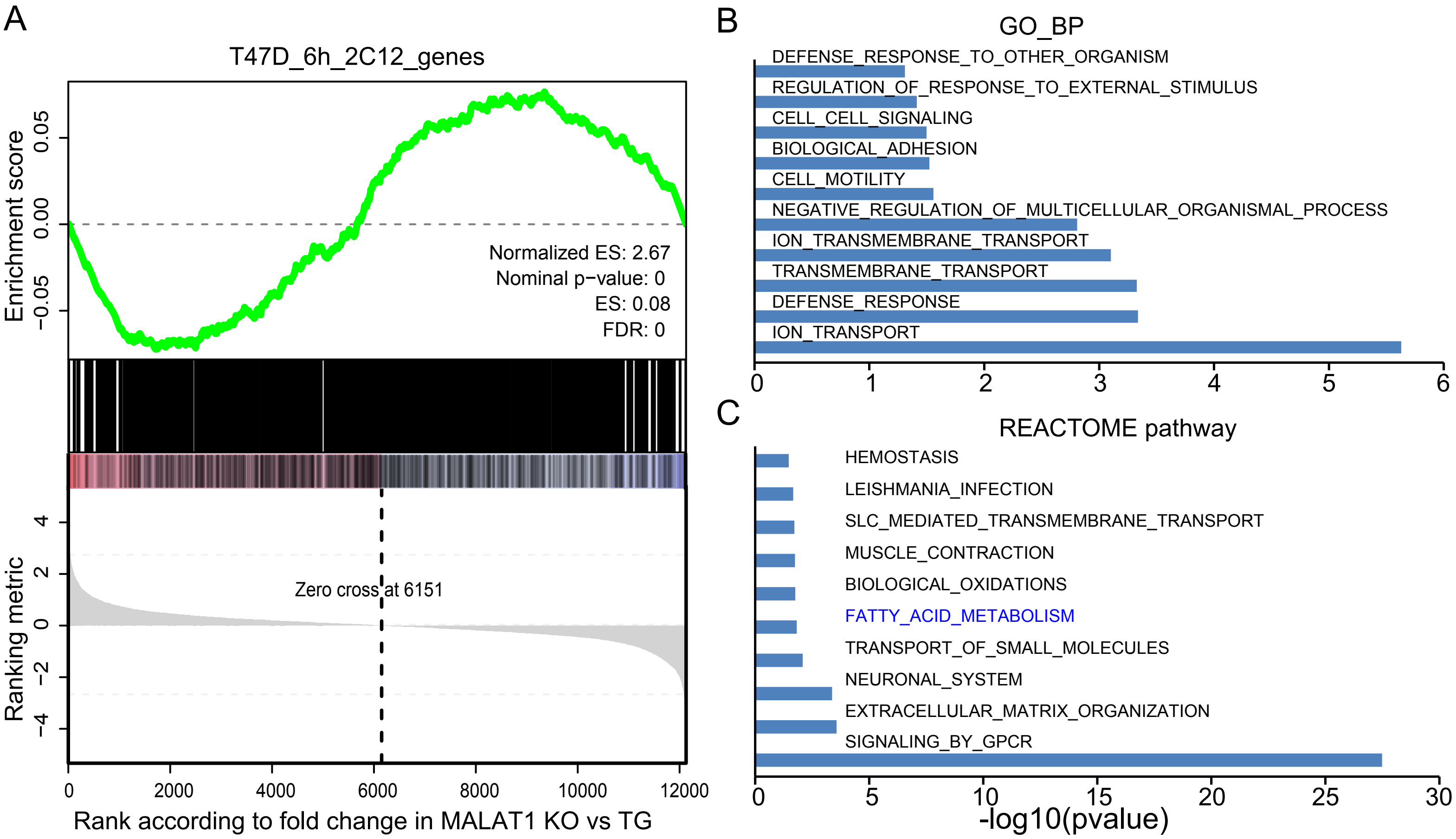

Figure S5. Function inference of MALAT1 via the CCN of T47D. (A) GSEA of RNAs in T47D_6h_2C12 cell to RNAs affected by MALAT1 knock out (KO). The top 10 functional terms from the (B) biological process of GO (GO_BP) and (C) REACTOME pathways enriched by RNAs in the CCN of T47D_6h_2C12 cell, as determined by the hypergeometric test. The interested terms are colored in blue.
