# Supplementary material for: Inferring Cell Subtypes and LncRNA Function by a Cell-Specific CeRNA Network in Breast Cancer
Source: Front Oncol. 2021 Apr 27;11:656675. doi: 10.3389/fonc.2021.656675 (PMC8111082; doi:10.3389/fonc.2021.656675)
Supplement: Supplementary file 7 [file Image_7.pdf]

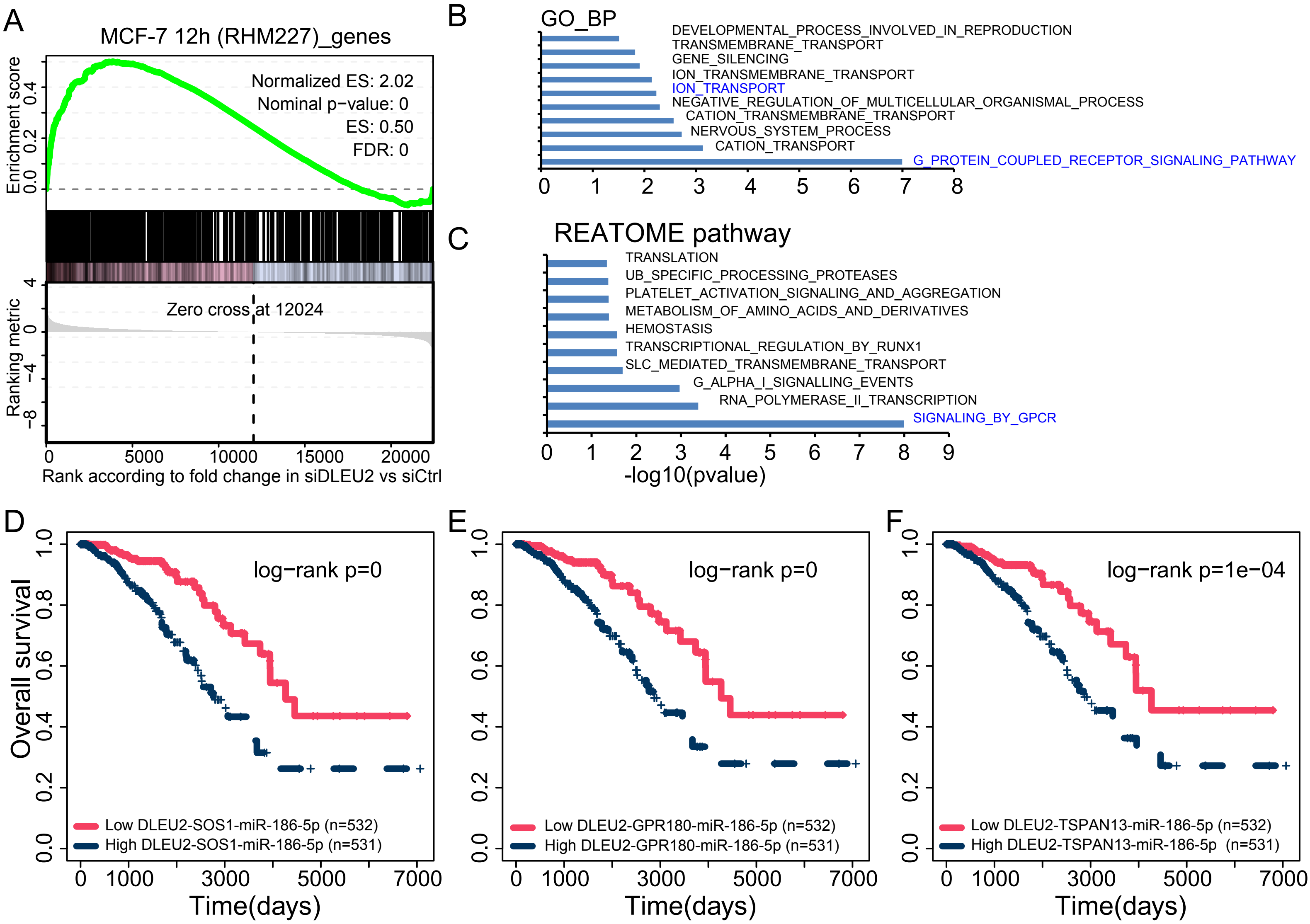

Figure S7. Function inference of DLEU2 via the CCN with negative correlation. (A) GSEA of RNAs in MCF-7 12 h (RHM227) cell to RNAs affected by siDLEU2. The top 10 functional terms from the (B) biological process of GO (GO_BP) and (C) REACTOME pathways enriched by RNAs in the CCN of MCF-7 12 h (RHM227) cell, as determined by the hypergeometric test. The interested terms are colored in blue. DLEU2 and its ceRNAs (D) SOS1, (E) GPR180, and (F) TSPAN13 have prognostic potential for breast cancer. The relapse-free (G) and metastasis-free survival analysis (H) performed by Kaplan-Meier Plotter for DLEU2.
