# Supplementary material for: Inferring Cell Subtypes and LncRNA Function by a Cell-Specific CeRNA Network in Breast Cancer
Source: Front Oncol. 2021 Apr 27;11:656675. doi: 10.3389/fonc.2021.656675 (PMC8111082; doi:10.3389/fonc.2021.656675)
Supplement: Supplementary file 8 [file Image_8.pdf]

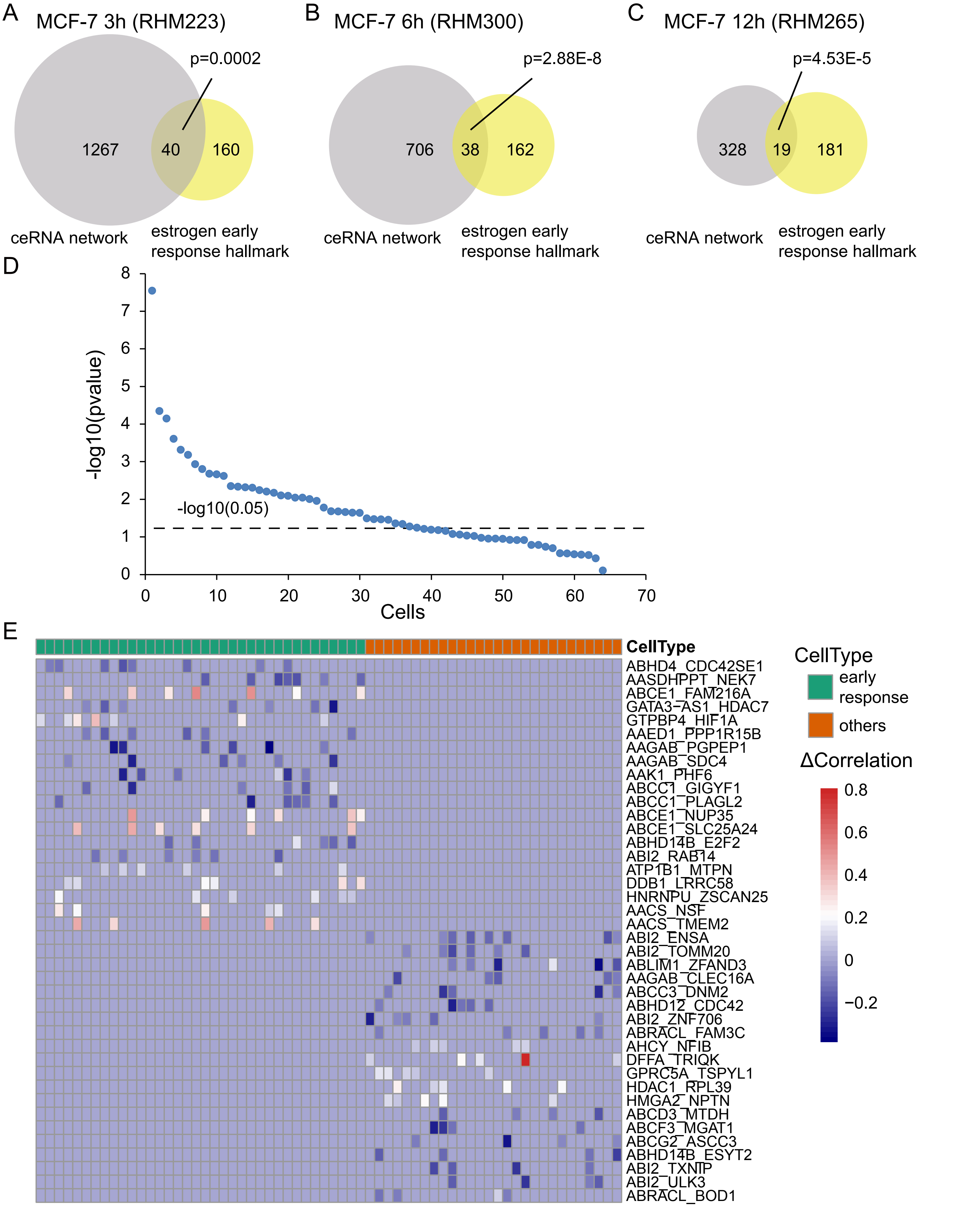

Figure S8. Cell subtypes inferred by the CCN with negative correlation. The RNAs in the CCN were enriched into estrogen early response hallmark, as determined by a hypergeometirc test. We showed significant enrichment of RNAs in the CCN of the (A) MCF-7 3 h (RHM223), (B) MCF-7 6 h (RHM300), and (C) MCF-7 12 h (RHM265) cells to the early response hallmarks. (D) The minus log10 transformed p-value calculated by a hypergeometirc test for all cells at 3 h, 6 h and 12 h. The dashed line represents the significance threshold, p = 0.05. (E) The heatmap of differential correlation in all cells that were classified into two subtypes: early-response cells vs. others. Blue represents loss of correlation in the “Perturbed network”, while red refers to the gain of correlation in the “Perturbed network”.
